# Dexamethasone Suppresses Oxysterol-Induced Differentiation of Monocytic Cells

**DOI:** 10.1155/2016/2915382

**Published:** 2016-05-31

**Authors:** Yonghae Son, Bo-Young Kim, Seong-Kug Eo, Young Chul Park, Koanhoi Kim

**Affiliations:** ^1^Department of Pharmacology, Pusan National University School of Medicine, Yangsan, Gyeongnam 50612, Republic of Korea; ^2^College of Veterinary Medicine and Bio-Safety Research Institute, Chonbuk National University, Iksan, Jeonbuk 54596, Republic of Korea; ^3^Department of Microbiology & Immunology, Pusan National University School of Medicine, Yangsan, Gyeongnam 50612, Republic of Korea

## Abstract

Oxysterol like 27-hydroxycholesterol (27OHChol) has been reported to induce differentiation of monocytic cells into a mature dendritic cell phenotype. We examined whether dexamethasone (Dx) affects 27OHChol-induced differentiation using THP-1 cells. Treatment of monocytic cells with Dx resulted in almost complete inhibition of transcription and surface expression of CD80, CD83, and CD88 induced by 27OHChol. Elevated surface levels of MHC class I and II molecules induced by 27OHChol were reduced to basal levels by treatment with Dx. A decreased endocytosis ability caused by 27OHChol was recovered by Dx. We also examined effects of Dx on expression of CD molecules involved in atherosclerosis. Increased levels of surface protein and transcription of CD105, CD137, and CD166 by treatment with 27OHChol were significantly inhibited by cotreatment with Dx. These results indicate that Dx inhibits 27OHChol-induced differentiation of monocytic cells into a mature dendritic cell phenotype and expression of CD molecules whose levels are associated with atherosclerosis. In addition, we examined phosphorylation of AKT induced by 27OHChol and effect of Dx, where cotreatment with Dx inhibited the phosphorylation of AKT. The current study reports that Dx regulates oxysterol-mediated dendritic cell differentiation of monocytic cells.

## 1. Introduction

Dexamethasone (Dx), a glucocorticoid, is widely used in treatment of chronic inflammatory and immunological diseases because it is a potent immune-suppressive and anti-inflammatory drug [1, 2]. Dx inhibits secretion of inflammatory mediators [3] and Th1 type immune response [4]. Dx also downregulates DC functions* in vivo* and* in vitro* [5]. Dx alters function of monocyte-derived dendritic cells (MoDCs) from cord blood [6] and inhibits maturation of dendritic cells (DCs) by redirecting differentiation of a subset of cells [7]. In addition, Dx inhibits the antigen presentation of DCs [8]. These reports indicate that Dx affects immunological responses by regulating differentiation and function of DCs.

DCs are potent antigen-presenting cells in the immune system and are critically involved in the initiation of primary immune responses and autoimmune responses that occur in diseases including atherosclerosis [9, 10]. DCs can be generated* in vitro* using monocytes. Human blood CD14^+^ monocytes differentiate into DCs when cultured in combination with granulocyte-macrophage colony stimulating factor (GM-CSF) and interleukin-4 (IL-4), and addition of tumor necrosis factor-*α* (TNF-*α*) induces maturation into CD83^high^-DC [10, 11]. Lipopolysaccharide (LPS), TNF-*α*, and calcium ionophore rapidly induce the differentiation of blood monocytes into DC-like cells [12]. THP-1, a human monocytic cell line, also differentiates into DCs. Treatment of THP-1 cells with cytokines and ionomycin results in differentiation into mature DCs (mDCs) [13]. 27-Hydroxycholesterol (27OHChol) also rapidly induces the differentiation of THP-1 cells into mDCs [14] with high expression of mDC-specific markers such as CD80, CD83, CD86, and CD88. However, there is no information regarding drugs that regulate 27OHChol-mediated differentiation into mDCs.

We were interested in the question whether Dx can affect DCs differentiation induced by 27OHChol. In the current study, we determine effects of Dx on expression of mDC markers and MHC molecules induced by 27OHChol, using THP-1 cells. In addition, we also examined whether Dx modulated expression of CD molecules, such as CD105, CD137, and CD166, which are associated with atherosclerosis.

## 2. Materials and Methods

### 2.1. Cell Culture and Reagents

THP-1 cells purchased from American Type Culture Collection (ATCC, Manassas, VA 20108) were maintained with RPMI 1640 containing 10% fetal bovine serum (FBS) in the presence of penicillin and streptomycin. 27OHChol was purchased from Research Plus, Inc. (Bayonne, NJ, USA). Dx was purchased from ENZO Life Science, Inc. (Farmingdale, NY). Fluorescein isothiocyanate- (FITC-) conjugated dextran (40 kDa) was purchased from Sigma-Aldrich (St. Louis, MO, USA). Primary antibodies were purchased from Santa Cruz Biotechnology (Santa Cruz, CA, USA). Alexa Fluor 488-conjugated secondary antibodies for FACS analysis were purchased from Invitrogen (Eugene, Oregon).

### 2.2. Dextran-FITC Uptake Assay

After incubation with Dx, 27OHChol, and PMA, THP-1 cells were resuspended in culture medium containing 1 mg/mL of FITC-conjugated dextran and incubated for 30 min at 37°C or 4°C (for background control). Cells were washed with cold phosphate buffered saline (PBS) containing 1% FBS, and flow cytometry was performed for analysis of uptake of FITC-dextran.

### 2.3. Reverse Transcriptase-Polymerase Chain Reaction (RT-PCR)

RT-PCR was performed as previously described [14]. In brief, total RNAs were reverse-transcribed for 1 h at 42°C with Moloney Murine Leukemia Virus reverse transcriptase, followed by PCR analysis. Glyceraldehyde-3-phosphate dehydrogenase (GAPDH) was amplified as a control with primers of 5′-GAGTCAACGGATTTGGTCGT (forward) and 5′-TGTGGTCATGAGTCCTTCCA (reverse). Primer pairs of CD molecules were designed using free online primer design tool primer 3. PCR products were visualized using ethidium bromide after electrophoresis on agarose gels.

### 2.4. Quantitative Real-Time Polymerase Chain Reaction

Quantitative real-time PCR was performed as previously described [15]. In brief, quantitative real-time PCR was performed in triplicate in 96-well plates containing SYBR Green PCR Master Mix and 10 pM forward primer and reverse primer for CD molecules and glyceraldehyde-3-phosphate dehydrogenase (GAPDH). The sequence of CD molecule primers was forward 5′-TGGTGCTGGCTGGTCTTTC and reverse 5′-CTGTGCCACTTCTTTCACTTCC (CD80); forward 5′-TCCTGAGCTGCGCCTACAG and reverse 5′-GCAGGGCAAGTCCACATCTT (CD83); forward 5′-GTGGTCCGGGAGGAGTACTTT and reverse 5′-GCCGTTTGTCGTGGCTGTA (CD88); forward 5′-CATCCTTGAAGTCCATGTCCTCTT and reverse 5′-GCCAGGTGCCATTTTGCTT (CD105); forward 5′-TCACTGCCTGGGGGCAGGAT and reverse 5′-GGCGGGGTCACAGAGGATGC (CD137); forward 5′-TCCTGCCGTCTGCTCTTCT and reverse 5′-TTCTGAGGTACGTCAAGTCGG (CD166). Primers for GAPDH were forward 5′-ATGGGGAAGGTGAAGGTCG and reverse 5′-GGGGTCAT TGATGGCAACAATA.

### 2.5. Flow Cytometric Analysis

After incubation with Dx and 27OHChol, THP-1 cells were harvested and incubated for 2 h at 4°C with antibodies against CD80, CD83, CD88, CD105, CD137, CD166, and major histocompatibility complex (MHC) class I and II molecules, followed by washing and incubation with fluorescent dye-conjugated secondary antibodies. Cells were washed and resuspended in 1% paraformaldehyde in PBS. Flow cytometry was performed for analysis of fluorescence.

### 2.6. ELISA

After incubation for 48 h with Dx and 27OHChol, the cells were analyzed using ELISA kit for phospho-AKT (R&D Systems, Inc. Minneapolis, MD, USA), following the manufacturer's instructions.

### 2.7. Statistical Analysis

Statistical analyses were performed using one-way ANOVA, followed by Tukey's multiple comparison tests, using GraphPad PRISM (version 5.0).

## 3. Results

### 3.1. Attenuated Expression of mDC Markers by Treatment with Dx

**Figure 1:**
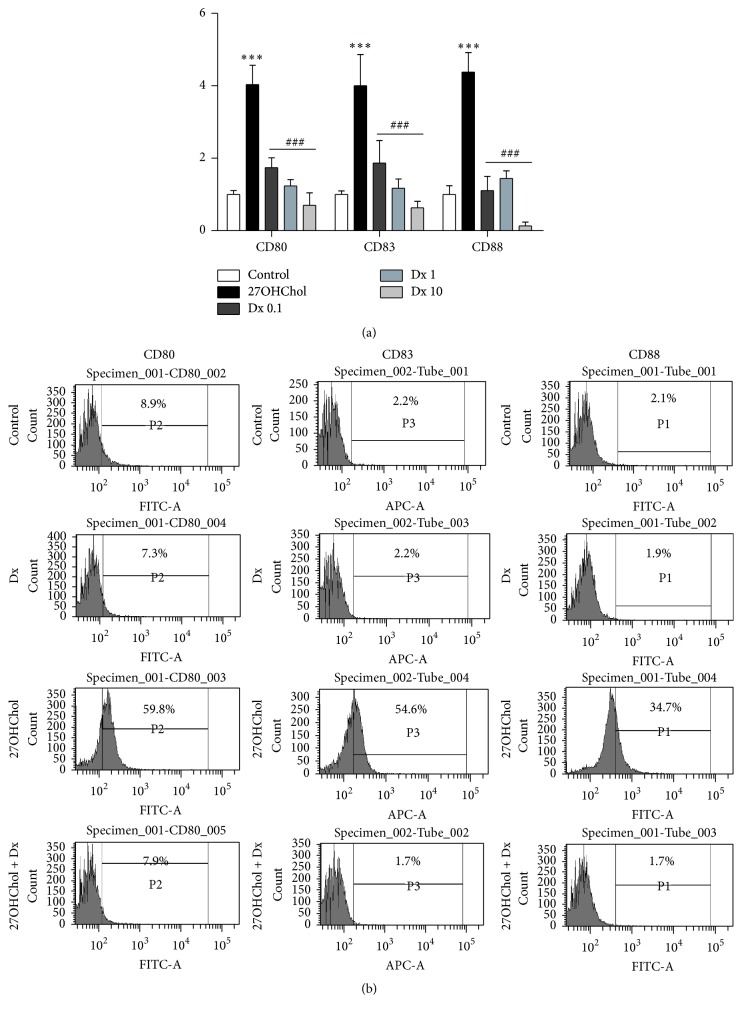
Effects of Dx on the transcription and surface expression of mDC markers induced by 27OHChol. (a) THP-1 cells (1 × 10^6^ cells/60 mm culture dish) were cultured for 48 h with 2.5 *μ*g/mL of 27OHChol with or without 0.1, 1, or 10 *μ*M of Dx. Transcription of CD80, CD83, and CD88 was analyzed by real-time PCR. Data are expressed as mean ± SD (*n* = 3 replicates/group). ^*∗∗∗*^ *P* < 0.001 versus control; ^###^ *P* < 0.001 versus 27OHChol. (b) THP-1 cells (1 × 10^6^ cells/60 mm culture dish) were cultured for 48 h in the presence of 2.5 *μ*g/mL of 27OHChol with or without 1 *μ*M of Dx. Cells were immunostained with antibodies against CD80, CD83, and CD88 and analyzed by flow cytometry. Results are representative of three independent experiments.

We determined expression of mDC markers to examine whether Dx affects differentiation of monocytic cells induced by 27OHChol. Treatment with 27OHChol resulted in significantly increased transcription of mDC markers CD80, CD83, and CD88, and addition of Dx resulted in their attenuated transcription, as determined by real-time PCR (Figure 1(a)). The levels of CD80, CD83, and CD88 were increased 4.03-, 4-, and 4.37-fold by treatment with 27OHChol, respectively, compared with unstimulated control, and the increases were reduced to be 1.74-, 1.86-, and 1.1-fold, respectively, by cotreatment with 0.1 *μ*M of Dx and almost completely inhibited to basal levels by treatment with 1 *μ*M and 10 *μ*M of Dx.

We also examined effects of Dx on surface expression of mDC markers (Figure 1(b)). In agreement with results of real-time PCR, flow cytometric analyses showed increases of CD molecules by treatment with 27OHChol. The percentages of control cells positive for CD80, CD83, and CD88 were 8.9%, 2.2%, and 2.1%, respectively, which increased to 59.8%, 54.6%, and 34.7%, respectively, by treatment with 27OHChol. However, the increases of mDC markers induced by 27OHChol were almost completely inhibited to control by treatment with Dx. Collectively, these results indicate that Dx reduced not only transcription but also surface expression of mDC markers in monocytic cells.

### 3.2. Attenuated Expression of MHC Molecules by Treatment with Dx

**Figure 2:**
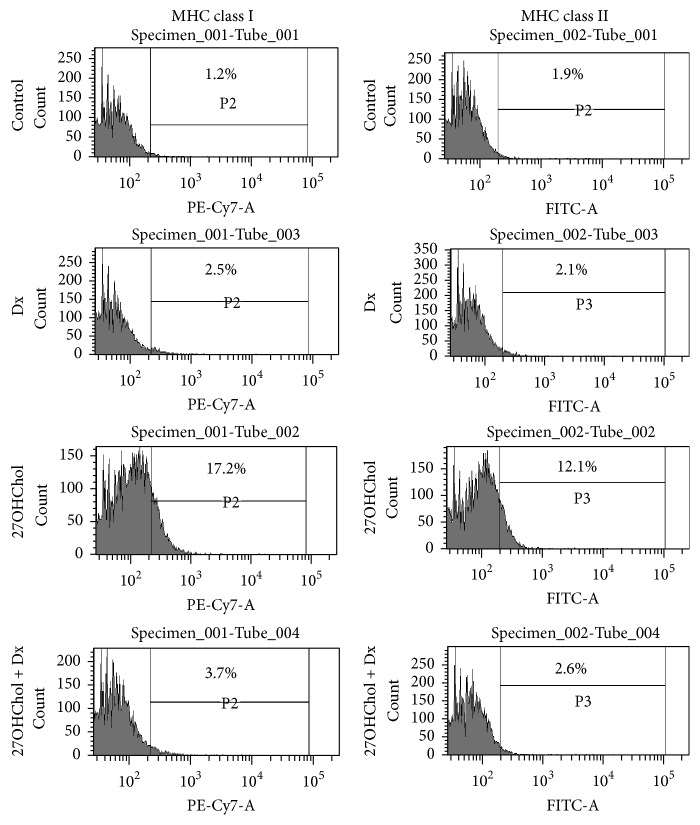
Effects of Dx on expression of MHC class I and II molecules induced by 27OHChol. After incubation for 48 h with 27OHChol (2.5 *μ*g/mL) with or without Dx (1 *μ*M), the stimulated THP-1 cells (1 × 10^6^ cells/60 mm culture dish) were immunostained for MHC classes I and II. Fluorescence was analyzed by flow cytometry. Results are representative of three independent experiments.

Flow cytometry was performed to examine whether Dx influenced expression of MHC class molecules (Figure 2). The percentage of control cells positive for MHC class I molecule was 1.2%, which showed a significant increase to 17.2% by treatment with 27OHChol. Similarly, the percentage of control cells positive for MHC class II molecule was 1.9%, which was increased to 12.1% by treatment with 27OHChol. However, the increased levels of MHC class molecules were reduced to control level by cotreatment with Dx. These results indicate that Dx inhibited surface expression of MHC molecules.

### 3.3. Recovered Endocytic Function by Treatment with Dx

**Figure 3:**
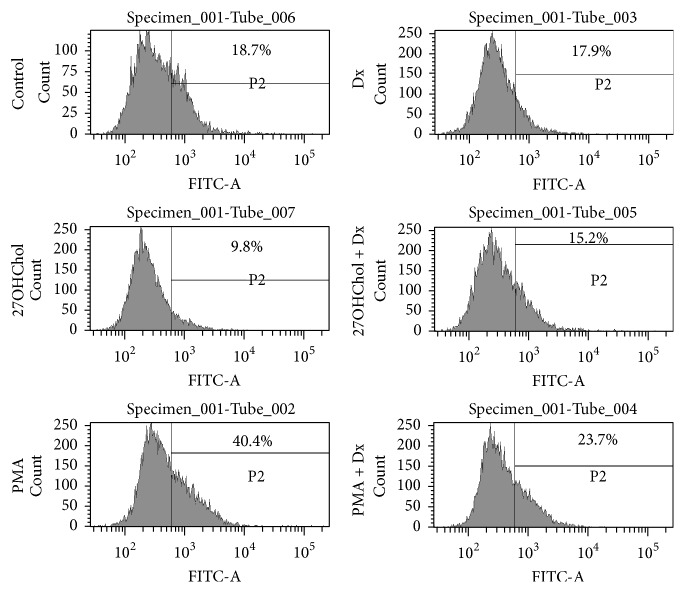
Effects of Dx on functional alteration of monocytic cells induced by 27OHChol. After incubation for 48 h with 27OHChol (2.5 *μ*g/mL) with or without Dx (1 *μ*M) and PMA (200 nM), THP-1 cells were treated with 1 mg/mL of FITC-conjugated dextran for 1 h. The cells were analyzed by flow cytometry. Results are representative of three independent experiments.

We performed an endocytosis ability test to determine whether Dx affected functional alteration induced by 27OHChol on monocytic cells (Figure 3). The percentage of control cells exhibiting endocytic activity was 19.5%, which was significantly reduced to 9.8% by treatment with 27OHChol and increased to 15.2% by cotreatment with Dx. Treatment with phorbol 12-myristate 13-acetate (PMA) resulted in an increase in percentage of cells exhibiting endocytic activity up to 40.4%, which was decreased to 23.7% by cotreatment with Dx. However, there was no change in endocytic function by treatment with Dx alone. These results indicate that endocytic function of monocytic cells modified by treatment with 27OHChol or PMA was recovered by Dx.

### 3.4. Attenuated Expression of Atherosclerosis-Associated CD Molecules by Treatment with Dx

**Figure 4:**
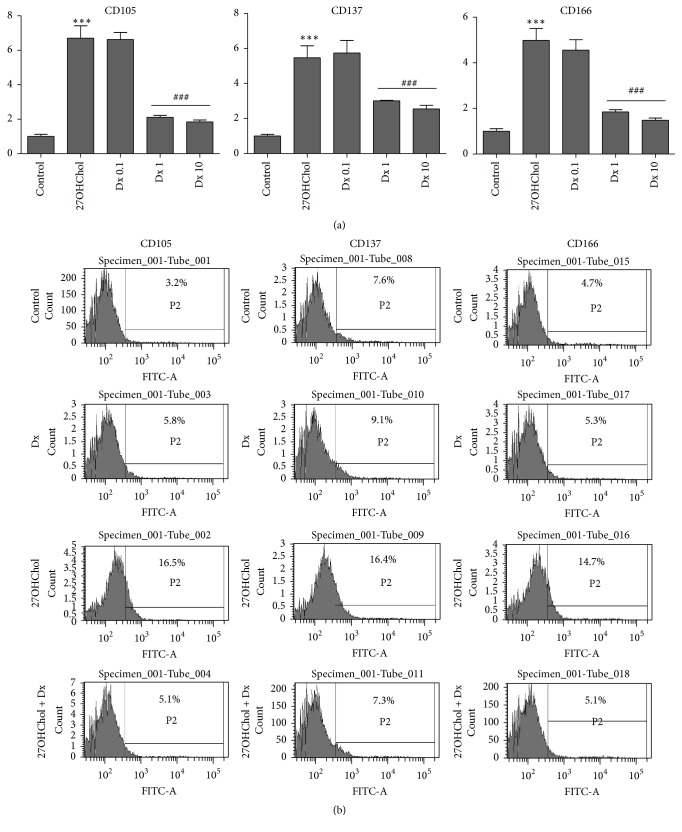
Effects of Dx on expression of atherosclerosis-associated CD molecules induced by 27OHChol. (a) After incubation with 27OHChol (2.5 *μ*g/mL) for 48 h with or without 0.1, 1, or 10 *μ*M of Dx, transcription of CD105, CD137, and CD166 was analyzed by real-time PCR. Data are expressed as mean ± SD (*n* = 3 replicates/group). ^*∗∗∗*^ *P* < 0.001 versus control; ^###^ *P* < 0.001 versus 27OHChol. (b) THP-1 cells (1 × 10^6^ cells/60 mm culture dish) were cultured for 48 h with 2.5 *μ*g/mL of 27OHChol with or without 1 *μ*M of Dx. The harvested cells were immunostained with antibodies against CD105, CD137, and CD166 and analyzed by flow cytometry. Results are representative of three independent experiments.

We examined whether Dx influenced expression of CD molecules CD105, CD137, and CD166, which are associated with atherosclerosis. Transcription of CD105, CD137, and CD166 was elevated, and addition of Dx resulted in decreased transcription of the genes (Figure 4(a)). Compared with control, the levels of CD105, CD137, and CD166 were increased 6.8-, 5.7-, and 4.9-fold, respectively, by treatment with 27OHChol, and the increases were reduced to basal levels by cotreatment with 1 *μ*M and 10 *μ*M of Dx. We also examined effects of Dx on surface expression of the molecules by flow cytometry (Figure 4(b)). The percentage of control cells positive for CD105 was 3.2%, which was increased to 16.5% by treatment with 27OHChol and was reduced to 5.1% by cotreatment with Dx. The percentage of CD137-positive control cells was 7.6%, which was increased to 16.4% by treatment with 27OHChol, which was reduced to 7.3% by Dx. The percentage of CD166-positive control cells was 4.7%, which was increased to 14.7% by treatment with 27OHChol and was reduced to 5.1% by Dx. These results indicate that expression of CD105, CD137, and CD166 induced by 27OHChol was significantly inhibited by cotreatment with Dx at the levels of transcription and protein.

### 3.5. Attenuated Phosphorylation of AKT by Treatment with Dx

**Figure 5:**
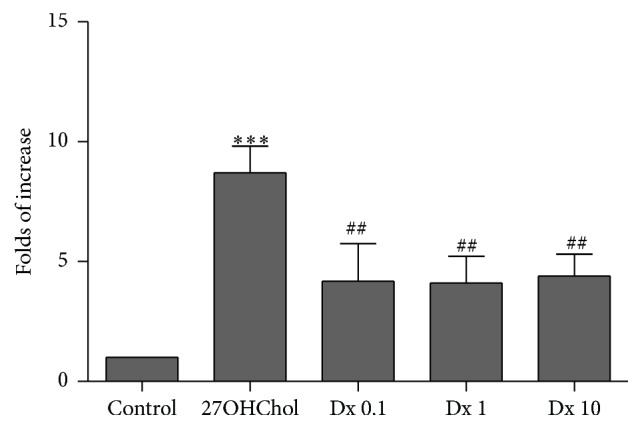
Effect of Dx on phosphorylation of AKT induced by 27OHChol. After incubation with 27OHChol (2.5 *μ*g/mL) for 48 h with or without 0.1, 1, or 10 *μ*M of Dx, lysates from the cells were analyzed with ELISA kit for p-AKT. Data are expressed as mean ± SD (*n* = 3 replicates/group). ^*∗∗∗*^ *P* < 0.001 versus control; ^##^ *P* < 0.05 versus 27OHChol.

To determine the 27OHChol-mediated pathway and the effects of Dx, we examined using ELISA kit for p-AKT (Figure 5). Phosphorylation of AKT was increased to be 8.7-fold by stimulation with 27OHChol, which was reduced to be 4.2-, 4.1-, and 4.4-fold, respectively, by treatment with 0.1, 1, and 10 *μ*M of Dx. This result showed that Dx affected the phosphorylation of AKT increased by 27OHChol.

## 4. Discussion

CD molecules overexpressed by 27OHChol not only are mDC-specific markers but also are involved in activation of T- and B-lymphocytes. CD80, also known as B7-1 or CD28 ligand, is a surface molecule involved in activation of B-lymphocytes [16]. CD83, a coactivator of T-lymphocytes, is required in development of CD4^+^ T cells [17]. CD88, known as C5a receptor, plays key roles in secretion of IL-12 and in Th2 type immune response [18]. Therefore, downregulated expression of CD molecules indicates that Dx will negatively regulate, in addition to differentiation of monocytic cells, activation of T-/B-lymphocytes. This idea is in line with previous studies reporting that Dx inhibited activation of T-/B-lymphocytes via enhanced apoptosis [19, 20]. These studies showed direct effects of Dx against activation of T and B cells whereas our result showed an indirect effect of Dx on activation of the cells. Activated T- and B-lymphocytes can promote atherosclerosis [21, 22]. It is possible that downregulated expression of CD80, CD83, and CD88 by Dx can affect pathogenesis of atherosclerosis. Further studies are needed in order to assess correlation between downregulated expression of CD molecules and atherosclerosis using an animal model treated with Dx.

MHC molecules play a role in antigen presentation captured by endo- or phagocytosis on the cell surface and, in contrast to monocytes or immature DCs (imDCs), are highly expressed on mDCs [9]. The expressed MHC molecules stimulate naïve T cells in secondary lymph nodes and induce adaptive immunity. Experimental evidence indicates that Dx can modulate expression of MHC molecules, thereby affecting antigen presentation. Dx downregulated expression of MHC class II on human monocytic cells stimulated with IFN-*γ* [23] and also inhibited differentiation into DCs and antigen presentation of DCs by MHC class II pathway induced by cytokines like GM-CSF and IL-4 [8]. The current study added new information to the previous reports that Dx reduces levels of surface MHC class I and II molecules induced by 27OHChol. The reduced expression of MHC I and II suggests that Dx can indirectly modulate activation of T cells through downregulation of MHC class molecules on 27OHChol-stimulated monocytic cells.

DCs capture antigens through endo- or phagocytosis. The captured antigens are digested in endo- or phagosome and presented by MHC molecules on cell surface. DCs show a different endocytosis activity in each stage [9, 24]. Antigen-uptake activity is high in immature DCs (imDCs) state but very low in mDC state. Dx-mediated recovery in endocytic functional change of monocytic cells induced by 27OHChol suggests that Dx can modulate antigen uptake and presentation of it on cell surface because antigen presentation is influenced by endocytosis activity. We consider that Dx-mediated recovery of endocytosis activity is associated with inhibition of 27OHChol-mediated differentiation. This idea is in agreement with the study by Piemonti et al., which showed that Dx recovered the endocytosis activity of MoDCs induced by GM-CSF and IL-4 through inhibition of differentiation from imDCs to mDCs [25]. Taken together, these findings indicate that Dx recovered changes of endocytic activity in monocytic cells differentiated by cytokines and oxysterol.

CD137 (known as 4-1BB), which is highly expressed in human atherosclerosis, promotes development of plaque inflammation [26], and its deficiency results in reduced atherosclerosis induced by hypercholesterolemia [27]. CD105 is overexpressed in atherosclerotic tissues [28, 29], and CD166 is a marker of atherosclerosis (USA patent number US8603829 B2). Therefore, the finding of upregulated expression of CD molecules by treatment with 27OHChol, which agrees with the previous study [14], indicates that 27OHChol is likely to increase their expression in atherosclerosis, thereby causing deterioration of the disease. In addition, decreased expression of the proatherogenic CD molecules will have beneficial effects by treatment with Dx because expression of CD molecules is correlated with severity of atherosclerosis. We think that the results of the current study provide a mechanism through which Dx induces suppressed atherosclerosis in an animal model of atherosclerosis [30].

We determined signaling pathways stimulated by 27OHChol. Some studies reported that 27OHChol promoted inflammations and immune response through estrogen receptor- (ER-) *α* [28, 31], and TLR4/NF-*κ*B signaling pathway was involved in the oxysterol-mediated responses [32]. We had studied that 27OHChol induced the upregulation of soluble CD14 (sCD14) and MMP-9 expression, AKT pathway was involved in the upregulation on monocytic cells [33]. However, which signaling pathways involved in the differentiation of monocytic cells induced by 27OHChol had not been reported. Our ELISA result suggests that 27OHChol increases phosphorylation of AKT, and Dx inhibited the increase. This data means that the 27OHChol-mediated differentiation mediated through AKT pathways. About signaling pathways involved in the differentiation, we perform more study.

Results of the current study suggest that Dx can modify immune responses by inhibiting differentiation of monocytic cells and expression of MHC molecules induced by 27OHChol and by reversing endocytic function. We propose that Dx disturbs immune response in a milieu rich in oxidatively modified cholesterol molecules like atherosclerotic plaques.
